# Significance of Haemodynamic and Haemostatic Factors in the Course of Different Manifestations of Cerebral Small Vessel Disease: The SHEF-CSVD Study—Study Rationale and Protocol

**DOI:** 10.1155/2013/424695

**Published:** 2013-01-02

**Authors:** Jacek Staszewski, Renata Piusińska-Macoch, Ewa Skrobowska, Bogdan Brodacki, Rafał Pawlik, Tomasz Dutkiewicz, Wiesław Piechota, Alicja Rączka, Kazimierz Tomczykiewicz, Adam Stępień

**Affiliations:** ^1^Clinic of Neurology, Military Institute of Medicine, Szaserow 128, 04-141 Warsaw, Poland; ^2^Department of Radiology, Military Institute of Medicine, Szaserow 128, 04-141 Warsaw, Poland; ^3^Clinic of Ophthalmology, Military Institute of Medicine, Szaserow 128, 04-141 Warsaw, Poland; ^4^Department of Laboratory Medicine, Military Institute of Medicine, Szaserow 128, 04-141 Warsaw, Poland

## Abstract

*Rationale.* This paper describes the rationale and design of the SHEF-CSVD Study, which aims to determine the long-term clinical and radiological course of cerebral small vessel disease (CSVD) and to evaluate haemostatic and haemodynamic prognostic factors of the condition. *Design.* This single-centre, prospective, non-interventional cohort study will follow 150 consecutive patients with different clinical manifestations of CSVD (lacunar ischaemic stroke, vascular dementia, vascular parkinsonism or spontaneous deep, intracerebral haemorrhage) and 50 age- and sex-matched controls over a period of 24 months. The clinical and radiological course will be evaluated basing on a detailed neurological, neuropsychological and MRI examinations. Haemodynamic (cerebral vasoreactivity, 24 h blood pressure control) and haemostatic factors (markers of endothelial and platelet dysfunction, brachial artery flow-mediated dilatation test) will be determined. *Discussion.* The scheduled study will specifically address the issue of haemodynamic and haemostatic prognostic factors and their course over time in various clinical manifestations of CSVD. The findings may aid the development of prophylactic strategies and individualised treatment plans, which are critical during the early stages of the disease.

## 1. Background

Effective therapeutic and preventive strategies in neurological diseases of the elderly are lacking. Cerebral small vessel disease (CSVD) is one of the most important and common vascular diseases of the brain. High morbidity rates are associated with CSVD; this disease leads to recurrent ischaemic and haemorrhagic strokes, gait disturbances, vascular dementia, and vascular parkinsonism [1, 2]. The course and prognosis of the disorder are not well known. Although patients with CSVD may exhibit white-matter lesion burdens on conventional MRIs that are almost identical, they may present clinically with a range of motor and cognitive deficits that is greatly varied. CSVD is related to vascular risk factors like hypertension, advanced age, and smoking; however, the direct pathophysiological mechanisms of the disease remain unclear [3]. Potential mechanisms include cerebrovascular risk factor-induced ischaemic cerebral changes and other nonspecific cerebral processes, such as generalised vascular disease or normal ageing [4]. The pathological components of CSVD probably include increased permeability of the blood-brain-barrier (BBB), enlargement of perivascular spaces, lacunar infarcts, white-matter lesions (WMLs), and microbleeds [5]. CSVD encompasses degenerative alterations in the vessel wall: arteriosclerosis/atherosclerosis, arteriolosclerosis, and lipohyalinosis which are assumed to be pathogenetically linked. Advanced age and chronic hypertension are the most important risk factors for CSVD. They induce hyperplastic arteriolosclerosis with deposits of fibrohyaline material, aneurysmal dilatation, and segmental arterial wall disorganisation of small, perforating intracerebral arteries which become susceptible to either occlusion or rupture leading to lacunar infarcts or spontaneous, deep hemorrhagic strokes, respectively. The reason why some vessel ruptures lead to major haemorrhage while others lead to microhaemorrhage is unknown. Pathological changes of intraparenchymal small arteries and arterioles are thought to represent the underlying vascular cause in combination with fluctuations of systemic blood pressure and altered cerebral blood flow autoregulation [6]. Lacunar strokes represent the focal manifestation of actually a diffuse abnormality of the small cerebral arterioles resulting in a state of chronic hypoperfusion of the white-matter, eventually resulting in degeneration of myelinated fibres as a consequence of repeated selective oligodendrocyte death. Multiple infarcts may also denote a more generalized phenomenon such as inflammation, oxidative stress, or disruption in the BBB which can manifest clinically as cognitive decline, dementia, or vascular parkinsonism [7]. Vascular cognitive impairment and vascular parkinsonism are not regular clinical and pathogenetic entities as they overlap with other diagnoses but the majority of patients present widespread microangiopathy-related cerebral damage [8]. Subcortical vascular cognitive impairment is now recognized to be the commonest form of vascular cognitive impairment, and it is recognized as subcortical vascular dementia in the International Classification of Diseases, 10th revision [9].

Markers of vascular inflammation and platelet reactivity are elevated in CSVD; however, their prognostic significance and role in different clinical manifestations of CSVD are not clear [10, 11]. It is unknown whether elevated inflammatory markers are a consequence of haemostatic disturbances in acute lacunar stroke or if they are secondary to generalised atherothrombosis or increased permeability of the BBB. The role of inflammation is speculated in vascular dementia whose incidence is influenced by the gene polymorphisms of the inflammatory mediators (interleukin 1, interleukin 6, TNF*α*, toll-like receptor 4, p-selectin, and C-reactive protein), nitric oxide release, and extracellular matrix (matrix metalloproteinases) [12]. Endothelial dysfunction may contribute to breakdown of BBB and impaired vessel reactivity and autoregulation [13].

Haemodynamic factors are known to play a key role in acute lacunar stroke. While they may predict stroke severity and prognosis, their role in various clinical presentations of CSVD has not been elucidated [14]. Impairments of cerebral autoregulation or vasomotor reactivity may precede and contribute to the onset of clinical dementia and development of WMLs, even in individuals who are asymptomatic, raising important research questions concerning the nature, severity, and causes of impaired cerebral autoregulation in CSVD [15, 16]. The relative importance of haemodynamic versus thromboembolic mechanisms in the pathogenesis of CSVD is difficult to ascertain. It is unknown whether these features provide prognostic information in CSVD that is more robust than the vascular factors traditionally used for this purpose. To date, no standardised prospective trials have been performed to assess the clinical and radiological course of the different manifestations of CSVD with respect to changes in vascular endothelium, thrombocyte function, and haemodynamic parameters.

The object of present study is to describe the long-term clinical and radiological course of most important manifestations of CSVD (lacunar and hemorrhagic stroke, vascular parkinsonism, and dementia) and to evaluate haemostatic and haemodynamic prognostic factors of the condition. Factors and mechanisms contributing towards variable phenotypic (clinical, functional, and neuroradiological) expression of CSVD linking cerebral blood flow and microvascular inflammation to the extent of white-matter damage can provide important mechanistic and therapeutic insights. The analysis of patients through a longitudinal study should also contribute to more advanced and conclusive evidence of the CSVD prognosis. The findings may aid in the development of prophylactic strategies and individualised treatment plans, which are critical during the early stages of the disease.

In the SHEF-CSVD Study (significance of hemodynamic and hemostatic factors in the course of different manifestations of cerebral small vessel disease) we investigate the role of hemodynamic and hemostatic factors in the course of CSVD prospectively.

To our knowledge, no other prospective cohort studies have been performed to associate these risk factors with different manifestations of CSVD. The research design and protocol of the SHEF-CSVD study are described.

This study was approved by the Medical Ethics Committee and is supported by the Polish Ministry of Science and Higher Education as a research project of the Military Institute of Medicine (Warsaw, Poland, study number N N402 473840). The authors have no conflict of interest to declare.

## 2. Methods/Design

### 2.1. Study Population

The symptoms of CSVD vary greatly and may overlap with other neurological conditions. They include acute lacunar syndromes and other acute neurological deficits, which are caused by spontaneous, deep haemorrhagic strokes. In addition, CSVD may present clinically as progressive chronic cognitive and/or motor disturbances, such as vascular dementia and vascular parkinsonism, respectively.

One hundred and fifty consecutive individuals referred to the Department of Neurology from October 2011, and 50 age- and sex-matched controls were selected as study candidates. Inclusion criteria for the study are as follows.

For CSVD patients:

- presenting with evidence of cerebral small vessel disease/white-matter lesions (WMLs) and lacunar or haemorrhagic infarct on neuroimaging (MRI),
- acute clinical symptoms due to nonembolic lacunar stroke according to the Oxford Community Stroke Project (OCSP) classification system [17, 18] (*n* = 50) (Table 1),
- or
- spontaneous deep intracerebral haemorrhage (*n* = 25),
- or
- chronic vascular parkinsonism after exclusion of other neurodegenerative conditions (Table 1) [19] (*n* = 25),
- or
- vascular dementia identified via the Modified Hachinski Ischemic Scale (score ≥ 7 points) and NINDS-AIREN (National Institute of Neurological Disorders and Stroke-Association Internationale pour la Recherche etl'Enseignement en Neurosciences) criteria [20, 21] (*n* = 50).

For control group:

- age, sex, and vascular risk factors-matched control group without known neuropsychiatric disease (*n* = 50).

For all patients:

- aged between 60 and 90 years AND,
- signed informed consent form.

Diagnosis of vascular dementia has been established based on widely used research settings, most sensitive Modified Hachinski Ischemic Scale, and most specific NINDS-AIREN set of criteria [22]. Eligible subjects, presenting with acute lacunar ischemic or haemorrhagic stroke, will be included in the study at least 2 weeks following the stroke to avoid acute effects on the outcomes measures.

Exclusion criteria for the study are

- non-CSVD-related WMLs (e.g., WMLs due to multiple sclerosis, migraine, CADASIL, vasculitis, and Lyme disease),
- large (>1.5 cm) subcortical infarcts or any cortical infarcts,
- history of cardioembolic stroke risk factors (e.g., atrial fibrillation and valvular heart disease) or carotid or intracranial artery stenosis ≥50% identified via ultrasound [23, 24],
- lacunar stroke treated with thrombolysis or any other previous ischaemic or haemorrhagic stroke,
- severe neurological deficits (modified Rankin Scale score ≥ 3 points, indicating at least moderate disability, and/or NIHSS ≥ 10 points), disturbed consciousness (NIHSS 1a ≥1), or severe dementia (MMSE < 12 points), requiring institutional care,
- psychiatric disorder interfering with cognitive testing or followup assessment (e.g., alcoholism),
- concomitant epilepsy, chronic heart failure (NYHA > II), malignant disease, and/or life expectancy of less than six months,
- significant visual and/or hearing impairment,
- MRI contraindications, such as claustrophobia,
- lack of reliable contact with study patients and their relatives during the study.

### 2.2. Aim

This single-centre, prospective, noninterventional cohort study will follow 150 consecutive patients with different clinical manifestations of CSVD (lacunar ischaemic stroke, vascular dementia, vascular parkinsonism or spontaneous deep, and intracerebral haemorrhage) and 50 age- and sex-matched controls over a period of 24 months. The study is designed to:

primary goals:

(1) assess the long-term clinical and radiological course of CSVD,
(2) evaluate haemostatic and haemodynamic prognostic factors that influence the clinical and radiological course of CSVD,

secondary goal:

(3) compare the clinical and radiological course between groups with CSVD, as well as between the CSVD and control groups.

All neurological, neuropsychological, and radiological assessments will be performed at baseline and at a 24-month followup examination. Baseline and followup assessments will be carried out by the same team of experienced investigators, who will be blinded to the clinical diagnosis of each subject.

The primary outcome measures used in this study will be the observed changes in neurological status, cognitive function, gait/balance, and parkinsonian signs in addition to the progression of WMLs visualised via MRI. Secondary outcome measures that will be used are vascular-induced causes of death and the occurrence of dementia, depression and vascular events.

### 2.3. Study Timeline

#### 2.3.1. Evaluation of Clinical Indicators of CSVD (Baseline and Followup Visits)

It is planned to enroll eligible patients within 12 months of recruitment. The progression of CSVD will be assessed based on findings from the neurological, neuropsychological, and radiological examinations performed at the baseline and 24-month followup visits.

#### 2.3.2. Cardiovascular Determinants

All patients will undergo a clinical evaluation, which includes an interview of past medical history and physical examination. Vascular risk factor profiles, which include information related to hypertension, diabetes mellitus, coronary artery disease, hypercholesterolemia, peripheral vascular disease, smoking, and alcohol intake, will be recorded for all participants at baseline and followup. Hypertension is defined as the mean systolic blood pressure ≥140 mm Hg, mean diastolic blood pressure ≥90 mm Hg, or self-reported use of antihypertensive drugs [25]. Diabetes mellitus is defined as a history of diabetes mellitus, fasting plasma glucose levels ≥7.0 mmol/L, or use of oral antidiabetic drugs or insulin [26]. Hyperlipidemia is defined as total cholesterol >5.0 mmol/L, low-density lipoprotein cholesterol >3.2 mmol/L, or the self-reported use of lipid-lowering drugs [27]. Tobacco use history will be calculated by multiplying the number of cigarette packs smoked per day by the number of years smoked. Participant height and weight will be measured to calculate body mass indexin kg/m^2^. The use of antiplatelet drugs, blood pressure-lowering medication, acetylcholinesterase inhibitors, and/or hypoglycaemic, hypolipaemic, and antiparkinsonian agents throughout the study will be recorded.

#### 2.3.3. Clinical and Neuropsychological Assessments

Health status as well as the presence and severity of disability will be assessed in all participants by the same two board-certified senior neurologists. The modified Rankin Scale (mRS) will be used to evaluate functional status, and the National Institutes of Health Stroke Scale (NIHSS) will be used to assess neurological impairment. To measure the ability to perform activities of daily living as well as disturbances in movement and gait, the Barthel Scale, the Unified Parkinson Disease Rating Scale (UPDRS), and the Berg Balance Scale will be used [28, 29]. 

These neuropsychological tests were chosen to investigate executive function and other cognitive domains that are typically impaired in CSVD. Assessments of cognitive and executive abilities that have been standardised and psychometrically validated will be utilised. The entire battery of tests includes measures of global cognitive function (the Mini Mental State Examination-Standardised) and various types of memory (the Wechsler Memory Scale-Revised, the Auditory-Verbal Learning Test, the Continuous Paired-Associate Word Learning Task, the Choynowski Memory Scale, the Benton Visual Retention Test-Revised and the Rey-Osterrieth-Complex Figure Test: Recall Administration) [30–32]. In addition, the battery includes tests of executive functions, which include attention and abstraction (the Wisconsin Card Sorting Test: Category Test, the Wechsler Adult Intelligence Scale: Digit Span and the Stroop Test) [33]. The remaining set of tests were selected to measure discursive and prosodic disorders (Bryan's Right Hemisphere Linguistic Battery), language deficits (the Boston Diagnostic Aphasia Examination, the Token Test), perceptual and graphomotor abilities (the Clock Drawing Test), depression (the Geriatric Depression Scale), severity of symptoms of dementia (the Clinical Dementia Rating), and disease burden (the Short Form Health Survey-SF-36) [34–40]. Z-scores will be generated for each cognitive test.

#### 2.3.4. MRI Protocol

The imaging protocol calls for multiple scan sequences, which include techniques performed at differing repetition and echo times (TR and TE, resp.). The protocol was designed to collect a series of T1-weighted images (TR = 600 ms, TE = 10 ms), T2-weighted images (TR = 5400 ms, TE = 68 ms), fast fluid-attenuation inversion recovery (FLAIR) images (TR = 8000 ms, TE = 120 ms), gradient-recalled echo (GRE) scans (TR = 850 ms, TE = 15 ms), and diffusion-weighted imaging (DWI) sequences (TR = 6000 ms, TE = 105 ms). These sequences will be performed using a GE Healthcare 1.5 Tesla scanner, and the slice thickness for all images will be set to 5 mm with no intersectional gaps.

The presence, number, and location (lobar, deep, or infratentorial) of various types of strokes will be recorded. Deep haemorrhagic strokes are defined as being in the late, subacute phase if hyperintense lesions on T1- and T2-weighted images are observed, or as being in the chronic phase if hypointensities are found on T1- and T2-weighted images. Lacunar infarcts are described as focal lesions between 3 and 15 mm in size, visualised as hyperintensities on T2 imaging, hypointense lesions on T1-weighted and hyperintense rim on FLAIR images. Scans from the imaging sequences will be assessed for subcortical and cortical infarcts (lesions ≥ 15 mm in size), microbleeds (focal areas < 5 mm in size, hypointense on GRE, and not representative of calcifications within the blood vessels), and enlarged Virchow Robin spaces (dilated perivascular spaces that appear hypointense on T1 or punctate or linear hyperintensities on T2 images) [41, 42]. DWI sequences will be used to detect and record acute and subacute infarcts.

White-matter lesions, which are visualised on T2 and PD/FLAIR images as ill-defined hyperintensities ≥5 mm, will be evaluated. The ARWMC Scale and the Fazekas Scale are used to quantify the severity of these lesions because they take the location, pattern, and extension of WMLs into account (Table 2) [43–45]. The ARWMC Scale scores five different regions in the right and left hemispheres separately: (1) the frontal area, (2) the parietooccipital area, (3) the temporal area, (4) the infratentorial area, and (5) the basal ganglia. The Fazekas Scale will be used to assess the presence of periventricular hyperintensities (PVH), observed as T2 and FLAIR hyperintense signal abnormalities in the periventricular areas, as well as deep WMLs. Global cerebral atrophy (GCA) will be assessed using FLAIR images and the GCA Scale, on which scores range from 0 to 3 [46]. All MRI scans will be obtained using the same scanner (GE Healthcare 1.5 Tesla scanner).

The complete set of baseline MRI variables of interest, which includes quantitative assessments of white-matter lesions, qualitative assessment of brain infarction, microbleeds, and cortical atrophy, will be compared with those obtained from followup MRI scans. Baseline and followup scans will be read independently and used to calculate change scores over time.

#### 2.3.5. Haemodynamic and Haemostatic Indicators (Baseline Visit)

Cerebral hemodynamic status is determined by assessing cerebral vasomotor reactivity (VMR) which provides information related to cerebral autoregulation and pulsatility indices (PIs); these indices reflect collateral circulation and increased vascular resistance that result from CSVD [47]. An experienced stroke neurologist will use a transcranial Doppler ultrasound (TCD) machine (Companion III TCD Systems, Nicolet) to record PI, VMR, and middle cerebral artery (MCA) velocity, which is a measure of mean CBF velocity. Based on our experience and the literature reports we expect that over 80% of measurements will be completed through the adequate acoustical temporal bone window [48]. VMR assessments will be performed after hyperventilation, the breath-holding test (BHT), and the acetazolamide (Diamox) test (ACT). Flow velocities and PIs will be measured in patients in a supine position in a quiet and breathing room air via transtemporal insonation of both MCA at a depth of 45–55 mm with a 2-MHz probes in a fixed position and averaged. The most powerful signal obtained during 10 cardiac cycles will be used to measure the mean flow velocities (MFVs). After 2 minutes of hyperventilation, administration of the BHT, resting MFVs, and time of breath holding (BHTT) will be recorded. The BHT will be performed according to the Markus and Harrison procedure, in which patients are instructed to hold their breath in one inspiration after a period of 4 minutes of normal breathing of room air. The ACT will be performed by intravenously injecting 10 mg/kg acetazolamide over 5 min. MCA MFVs are recorded before infusion and 15 minutes after it has taken place [49, 50]. Blood pressure and heart rate are simultaneously monitored during the procedure; an increase in either of these parameters of >20% leads to excluding the data from analysis. All tests will be performed separately with breaks of 4 minutes between test administrations [51–53]. The following variables are automatically calculated from the tests: (1) vasomotor reserve VMRr = [(MFV_BHT_ − MFV_hyperventilation_)/MFV_rest_]∗100, (2) breath holding index BHI = {[(MFV_BHT_ − MFV_rest_)/MFV_rest_]/BHTT}∗100, and (3) vasomotor reactivity VMR = [(MFV_ACT_ − MFV_rest_)/MFV_rest_]∗100.

Assessments of carotid intima-media thickness and atheromatous stenosis of the extracranial arteries will be performed according to previously validated criteria by colour-flow B-mode Doppler ultrasonography (Loqic P6, GE Healthcare) [54]. 

Ischemia-induced brachial artery flow dilatation, which reflects endothelial function, will be assessed. The procedure for this technique includes ultrasound scanning of the brachial artery at rest (baseline), as well as during hyperaemia, which will be induced by 5 minutes of inflating and deflating a sphygmomanometer cuff placed around the forearm distal to the ultrasound site [55]. Stress is created by increased blood flow following transient ischemia and induces the release of nitric oxide (NO), which results in local arterial vasodilatation. Flow-mediated dilatation (FMD) is estimated as the percent increase in vessel diameter from baseline to maximum vasodilation, which occurs during hyperaemia [56].

Twenty-four-hour ambulatory blood pressure (BP) monitoring (ABPM) will be performed using a portable noninvasive oscillometric and auscultatory device (Schiller MT-300). Measurements over a 24-hour period are recorded every 15 minutes during daytime hours (07.00 to 23.00) and every 30 minutes during nighttime hours (23.00 to 7.00). For recordings to be considered valid, a minimum of 15 daytime and 8 nighttime measurements is required. The mean 24-hour day and night systolic BP (SBP), diastolic BP (DBP), pulse pressure (PP), relative nocturnal dipping (SBP dipping), and mean heart rate will be calculated and recorded automatically. TCD and BP monitoring will be performed on the same day.

For all participants, serum cholesterol, HDL, LDL, triglycerides, glucose, HbA1c, uric acid, and albumin levels will be assessed. Evaluation of the endothelial and thrombocyte functions include: hsCRP, ICAM 1, PAI-1, thrombomodulin, homocysteine, d-dimers, fibrinogen, IL-1, anticardiolipin antibody, vit.B12, MPV, PLT, and sP-selectin. These functions are measured with commercially available ELISA kits according to the manufacturer's instructions. 

Platelet aggregation in whole blood will be evaluated with multiple electrode platelet aggregometry (multiplate analyser, Dynabyte, Munich, Germany) [57]. Blood is collected in test tubes containing the anticoagulant hirudin (25 *μ*g/mL) (Sarstedt, Germany). We will use the following aggregation agonists: arachidonic acid (AA), adenosine diphosphate (ADP), and thrombin receptor agonist peptide (TRAP), all of which are supplied by the manufacturer of the aggregometer. Blood for testing will be collected in the morning from fasting patients, and assays will be performed within 2 h of blood collection. Assessments of aggregation include evaluation of the area under the curve (AUC) of the aggregation plot, the degree of aggregation (AGR), and aggregation velocity (VAGR). Aggregation is determined twice in each sample, and the mean value is calculated if the two values differ by less than 10%. In the event that the values differ by 10% or more, the aggregation assays will be repeated.

#### 2.3.6. Other Clinical Indicators (Baseline Visit)

Retinal vessels can be visualised noninvasively and will be used to assess systemic vascular damage. The presence of retinal vessel abnormalities will be analysed using digitised fundus transparencies with a semiautomated system [58]. After dilation of the pupils with 1% tropicamide, retinal photographs centred on the optic disc will be taken off each eye using a nonmydriatic digital camera. The calibre of retinal arterioles and venules is measured from the photograph using a validated computer-assisted method. The retinal arteriole/venule ratio (AVR) is calculated by dividing the mean arteriolar calibre by the mean venular calibre.

#### 2.3.7. Followup

Two years after taking the baseline measurements, all participants will be contacted for prospective assessment of potential outcome events using the same instruments as at baseline visit including neurological (mRS, NIHSS, the Barthel Scale, UPDRS, and the Berg Balance Scale), neuropsychological (full battery of previously described tests), and radiological (MRI) examinations. Recurrent strokes and/or death due to cardiovascular reasons will be noted. If these assessments cannot be administered in person, researchers will contact the patient or his/her caregiver via phone. The patient's functional state will be assessed according to the mRS in cases requiring a phone call. 

## 3. Statistical Analysis

Recent studies demonstrated a high rate of WMLs progression (36%–75%/3 years) detected with repeated MRI even in otherwise healthy older populations [59]. Advanced age, WML severity, and presence of lacunar brain infarcts at baseline were strongly associated with lesion progression independent of other cardiovascular risk factors [60]. Based on study inclusions criteria which required documented WMLs at baseline MRI, it is expected that our study population has a relatively high degree of WMLs and high risk of progression. A sample size analysis was performed to determine the minimum number of patients required. The minimum effect size was 10%. Accounting for a maximum estimated 10% loss to followup, a total of 150 patients with CVSD and 50 from CG will be required to test the null hypothesis with an *α* value of 0.05 and a power of 0.8. Based on the hospital data set and estimated annual number of patients admitted due to CSVD we decided to establish unequal CSVD groups: with lacunar stroke (*n* = 50), vascular dementia (*n* = 50), deep hemorrhagic strokes (*n* = 25), and vascular parkinsonism (*n* = 25). Student's *t*-test and chi-squared tests will be used to make between-groups comparisons of continuous and categorical variables. Log-transformations will be used to correct for nonnormality of the data. Demographic data, vascular risk profiles, and hemodynamic data of the study groups will be compared by Student's *t*-test and ANOVA. The degree of neurological improvement or deterioration will be assessed via comparing the baseline and followup results. Pearson's correlation coefficients and ANOVA will be used to correlate haemodynamic data and neurological status at baseline and followup time points. The association between WML severity and haemodynamic and haemostatic data and cognitive, motor, and balance function will be assessed using linear regression models. Changes in MRI parameters neuropsychological and neurological data between the 2 time points will be assessed via paired *t*-tests for parametric data or the Wilcoxon signed-rank test for nonparametric data. Percent difference for each of the parameters will be calculated. Multivariate analysis that is adjusted for potentially confounding subject-relevant variables (age, sex, and vascular risk factor profile) will be performed to determine which haemostatic and haemodynamic factors are most useful measures of association to predict changes in neurological status, neuropsychological function, and severity of WMLs at baseline and followup.

## 4. Summary

Despite conceivable advances in our understanding of the natural history of WMLs over the past few years, several research questions need to be resolved. Numerous studies have shown that WMLs are related to vascular risk factors. The exact diagnosis of CSVD is, however, still not well known and requires systematic evaluation of potentially relevant clinical and other phenotypic features. It has been suggested that a prothrombotic state and endothelial activation are possible causal factors but recent neuropathology study did not confirm that relation [61–63]. It is also unknown how endothelial activation and likely consequent dysfunction mediate its effect. The “gold standard” for confirming the clinical diagnosis of vascular parkinsonism and dementia is also not established, and the literature reports that the associations between WMLs and cognitive and motor decline are rather weak [64]. The “optimal” treatment with regard to primary and secondary prevention of CSVD is not clear [65]. The SHEF-CSVD study is a large, prospective study that examines the significance of haemodynamic and haemostatic prognostic factors and their course over time in CSVD. To the best of our knowledge, no prospective cohort studies that investigate haemodynamic and haemostatic factors in differing manifestations of CSVD have been performed. Strengths of the SHEF-CSVD study include the prospective nature of the study; two protocols will be completed at baseline and 24-month followup. Additionally, all of these protocols will take place in one research centre, and the same researchers will collect all the data for the study using the same scanner. Furthermore, the tests and radiologic protocols chosen for this study are widely accepted and have been proven to be specific and sensitive in populations with WMLs. Still, some methodological issues should be considered. First, there is a possibility of selection bias. Second, we use an MRI semiquantitative visual measurement tool, which gives less precise estimates of lesion progression than a volumetric measurement. Larger number of participants with wider range of population groups and the ability to compare results among different sites in multicentre trial would potentially increase the generalizability of the study but it was not economically nor logistically feasible for our study group. The study has, however, the potential to unravel the functional and neuropsychological consequences of WMLs progression with regard to different hemostatic and hemodynamic factors which could serve as potential therapeutic targets. With the aging of the society, the importance of early diagnosis and prophylactic treatment of CSVD should be greatly noticed. 

## Figures and Tables

**Table 1:** Diagnostic criteria for lacunar stroke, deep intracerebral haemorrhage, chronic vascular parkinsonism, and vascular dementia.

Lacunar stroke	Deep intracerebral haemorrhage	Chronic vascular parkinsonism	Vascular dementia	Points
(i) One of the recognised lacunar syndromes (unilateral motor/sensory signs involving the whole of at least 2 of the 3 bodyparts (face, arm, and leg))	(i) Spontaneous haemorrhage confirmed by CT scan in the thalamus, basal ganglia or pons, presumably due to CSVD	(i) Subacute or acute onset	(i) Abrupt onset	2
(ii) No disturbances of consciousness or cortical functions	(ii) Exclusion of other causes (e.g., trauma, altered hemostasis, haemorrhagic necrosis, cerebralvenous thrombosis, rupture of an aneurysm or arteriovenous malformation, other arteriopathies including cerebral amyloid angiopathy, moyamoya disease)	(ii) Stepwise progression of symptoms	(ii) Stepwise progression of symptoms	1
(iii) a small hyperintense, sharply marginated subcortical lesion ≥3 mm <15 mm in diameter on T2-weighted MRI, which is compatible with the clinical findings listed above		(iii) Presence of vascular risk factors	(iii) Fluctuating course of disorder	2
		(iv) Predominant signs of lower body parkinsonism	(iv) Nocturnal confusion (sundowning)	1
		(v) Lack of significant clinical response to L-Dopa	(v) Relative preservation of personality	1
		(vi) Typical radiological findings of CSVD	(vi) Depression	1
			(vii) Somatic complaints	1
			(viii) Emotional lability	1
			(ix) History of hypertension	1
			(x) History of stroke	2
			(xi) Evidence of associated atherosclerosis	1
			(xii) Focal neurological symptoms	2
			(xiii) Focal neuroradiological findings	2

**Table 2:** Semiquantitative assessment of the severity of white matter damage in visual rating scales.

ARWMC rating scale	Fazekas scale	
*White matter lesions* No lesions (including symmetrical, well-defined caps or bands) Focal lesions Beginning of confluence of lesions Diffuse involvement of the entire region, with or without involvement of U fibres	*Periventricular hyperintensities (PVH)* Absent Caps or pencil-thin lining Smooth halo Irregular PVH extending into the deep white matter	*Deep white-matter hyperintensities* Absent Punctate foci Beginning confluence of foci Large confluent areas
*Basal ganglia lesions* No lesions Focal lesion (≥5 mm) >1 focal lesion Confluent lesions		
